# The Emerging Link Between the Complement Cascade and Purinergic Signaling in Stress Hematopoiesis

**DOI:** 10.3389/fimmu.2018.01295

**Published:** 2018-06-05

**Authors:** Mariusz Z. Ratajczak, Mateusz Adamiak, Magda Kucia, William Tse, Janina Ratajczak, Wieslaw Wiktor-Jedrzejczak

**Affiliations:** ^1^Stem Cell Institute at James Graham Brown Cancer Center, University of Louisville, Louisville, KY, United States; ^2^Department of Regenerative Medicine, Center for Preclinical Research and Technology, Warsaw Medical University, Warsaw, Poland; ^3^Department of Hematology Warsaw Medical University, Warsaw, Poland

**Keywords:** complement cascade, ATP, mannan-binding lectin, sterile inflammation, purinergic signaling

## Abstract

Innate immunity plays an important role in orchestrating the immune response, and the complement cascade (ComC) is a major component of this ancient defense system, which is activated by the classical-, alternative-, or mannan-binding lectin (MBL) pathways. However, the MBL-dependent ComC-activation pathway has been somewhat underappreciated for many years; recent evidence indicates that it plays a crucial role in regulating the trafficking of hematopoietic stem/progenitor cells (HSPCs) by promoting their egress from bone marrow (BM) into peripheral blood (PB). This process is initiated by the release of danger-associated molecular patterns (DAMPs) from BM cells, including the most abundant member of this family, adenosine triphosphate (ATP). This nucleotide is well known as a ubiquitous intracellular molecular energy source, but when secreted becomes an important extracellular nucleotide signaling molecule and mediator of purinergic signaling. What is important for the topic of this review, ATP released from BM cells is recognized as a DAMP by MBL, and the MBL-dependent pathway of ComC activation induces a state of “sterile inflammation” in the BM microenvironment. This activation of the ComC by MBL leads to the release of several potent mediators, including the anaphylatoxins C5a and _desArg_C5a, which are crucial for egress of HSPCs into the circulation. In parallel, as a ligand for purinergic receptors, ATP affects mobilization of HSPCs by activating other pro-mobilizing pathways. This emerging link between the release of ATP, which on the one hand is an activator of the MBL pathway of the ComC and on the other hand is a purinergic signaling molecule, will be discussed in this review. This mechanism plays an important role in triggering defense mechanisms in response to tissue/organ injury but may also have a negative impact by triggering autoimmune disorders, aging of HSPCs, induction of myelodysplasia, and graft-versus-host disease after transplantation of histoincompatible hematopoietic cells.

## Introduction

The basic function of innate immunity is to alarm the organism of an infection or tissue/organ injury in order to launch an appropriate response. An important part of this response is the release or mobilization of effector cells, such as granulocytes, monocytes, and lymphocytes, from bone marrow (BM) and other hemato-lymphatic organs into peripheral blood (PB) and the lymphatics and will be involved in eliminating invading pathogens (1–4). In parallel, hematopoietic stem/progenitor cells (HSPCs) are also released, which locally supply mature granulocytes or dendritic cells by clonal expansion of progenitors in the damaged tissues (3–6). Moreover, in addition to HSPCs, other types of stem cells are also released at a much slower pace into the circulation, including (i) mesenchymal stem cells (MSCs), (ii) endothelial progenitor cells (EPCs), and (iii) rare, primitive very small embryonic-like stem cells (VSELs). If needed, all of these stem cells may be involved in repair mechanisms in damaged tissues (4, 7–9).

Bone marrow is a semi-solid tissue spread within the spongy or cancellous portions of bones and contains hematopoietic “red marrow,” which is the most important source of cells circulating in PB and in the lymphatics (1–6). The estimated total mass of BM tissue in an average human being is as much as 6 pounds. This dynamic organ daily produces approximately 5 × 10^11^ erythrocytes, leukocytes, monocytes, and platelets, which enter the systemic circulation by crossing the BM–PB barrier *via* a permeable vasculature of small-vessel sinusoids within the medullary cavity. As mentioned above, BM is also the birthplace of stem cells that circulate in PB (1–6). While stem cells reside in stem cell niches, which are located around small vessels (endothelial niches) and in contact with osteoblasts lining trabecular bones in BM (osteoblastic niches), granulocytes, monocytes, and other types of maturing hematopoietic cells (mostly erythroblasts) occupy the entire volume of the hematopoietic microenvironment (1, 10–13).

Under steady-state conditions, maturing erythrocytes, leukocytes, monocytes, and platelets enter the PB to replace blood cells that have a limited half-life along with stem cells that are patrolling peripheral tissues, keeping the stem cell pool at distant locations of the hematopoietic microenvironment in balance (1–6). This balance may rapidly change in response to inflammation and tissue/organ damage when more cells need to be released into the circulation. This requires intensification of hematopoiesis in the BM microenvironment to supply more blood cells, while at the same time more stem cells are released from their BM niches (1–4). Increased release of cells from BM occurs also in clinical settings after pharmacological mobilization of HSPCs in response to administration of certain pro-mobilizing drugs, such as granulocyte colony-stimulating factor (G-CSF), CXCR4 receptor antagonists, or some chemokines (growth-regulated protein beta, Gro-β) (14–16).

In this review, we will present the accumulated evidence that a major orchestrator in the release of cells from BM into PB is the complement cascade (ComC), which induces a “sterile inflammation” state in the hematopoietic microenvironment (17, 18). The ComC can be activated by the classical, alternative, or mannan-binding lectin (MBL) pathways. Recent evidence indicates that acute activation of the MBL pathway of ComC activation plays the most important role in the release of cells from BM in response to tissue/organ injury, pathogens, and certain pro-mobilizing drugs (17–19). On the other hand, chronic activation of the MBL pathway is most likely an important element in BM aging and myelodysplasia (20–23). This pathway also likely contributes based on some clinical observations to induction of graft-versus-host disease (GvHD) after histoincompatible hematopoietic transplantation (24–27).

What is important for the topic of this review is that the MBL pathway of ComC activation is triggered by danger-associated molecular patterns (DAMPs) (28–33). Adenosine triphosphate (ATP) is one of the most important members of this family of molecules. However, it is well known that this ubiquitous intracellular molecular energy source, when secreted from cells, becomes an important signaling molecule and mediator of purinergic signaling (34–36). The release of ATP from cells in the BM microenvironment provides a molecular basis, involving activation of the ComC, for the link between purinergic signaling and activation of the innate immune response.

In this review, we will focus on the role of this ATP-mediated link between purinergic signaling and innate immunity in BM stem cell homeostasis, mobilization, and aging as well as in certain pathological conditions, including myelodysplasia and GvHD. Because of space limitation, our short review will not discuss several pathologies related to (i) chronic activation of ComC seen in paroxysmal nocturnal hemoglobinuria or atypical hemolytic-uremic syndrome, (ii) coagulation consequences due to interaction between ComC and coagulation cascade (CoaC), and (iii) ComC activation related to some cases of leukopenia or thrombocytopenia.

## Retention of HSPCs in BM and Their Release Due to Activation of Innate Immunity

Hematopoietic stem/progenitor cells reside in BM niches, and some important mechanisms mediating their BM retention have already been identified (1, 10–13, 37). The most important mechanisms include (i) the interaction between the chemokine receptor CXCR4 expressed on the surface of HSPCs and its specific ligand, the α-chemokine stromal-derived factor 1 (SDF-1) expressed by cells in stem cell niches and (ii) the interaction between the integrin receptor known as very late antigen 4 (VLA-4), which is expressed by HSPCs, and its ligand in stem cell niches, vascular adhesion molecule 1 (VCAM-1) (1–4). What is important for the retention process is that both receptors, CXCR4 and VLA-4, are located in special cell membrane domains enriched for cholesterol and glycosyl phosphatidylinositol anchor protein (GPI-A) known as membrane lipid rafts (38, 39). Of note, the same membrane lipid rafts also contain the cell-surface proteins CD55 and CD59 that regulate complement activity (29, 30, 40). Accumulating evidence indicates that the structural integrity of membrane lipid rafts on the surface of HSPCs is important for their retention in BM niches (38, 39). A significant role in retention of HSPCs in BM niches is also played by the third protein component (C3) of the ComC, as its cleavage fragments, C3a and _desArg_C3a, promote incorporation of CXCR4 and VLA-4 into membrane lipid rafts (41). In addition, the interaction of C3a with C3aR, which is expressed on the surface of HSPCs, directly increases adhesion of HSPCs in the BM microenvironment (41).

Results from our group also indicate that the release of HSPCs from BM niches into PB in response to administration of pharmacological mobilizing agents, as well as to mediators released during tissue/organ injury, is triggered by activation of the ComC (4, 18, 19). The same mechanism plays a pivotal role in the release of other types of stem cells, including MSCs, EPCs, and VSELs. In support of the regulatory involvement of the ComC in the retention of HSPCs in BM niches, we have already demonstrated that, while blockage of C3aR on the surface of HSPCs promotes the mobilization process (42), cleavage of the fifth protein component (C5) and release of C5a and _desArg_C5a anaphylatoxins is crucial for egress of HSPCs into PB (43). Mice that were deficient in C5 and C5aR turned out to be poor mobilizers (43). We propose that the proximal and distal part of ComC regulates retention of HSPCs in the BM microenvironment in opposite manner (18, 42, 43). While activation of the proximal part of this cascade *via* C3 cleavage fragments promotes retention of cells in BM, activation and cleavage of C5 have the opposite effect, as C5 cleavage fragments promote their egress (4, 18, 42, 43). This demonstrates a fine-tuned ComC-mediated mechanism in auto-controlling this process.

At the beginning of our work on the role of the ComC in regulating trafficking of HSPCs, we posed the basic question of which of the ComC-activation pathway (classical, alternative, or MBL) plays a crucial role in triggering egress of cells from BM. Initially, we considered the involvement of the classical pathway. To our surprise, however, mice deficient in the C1q component of classical pathway activation turned out to be good mobilizers in response to administration of the most commonly used HSPC mobilizing agent, G-CSF (44). Therefore, we shifted our attention to the MBL pathway of ComC activation and performed mobilization studies in MBL-KO animals (19). In our experiments, MBL-KO or wild-type (WT) control mice were mobilized with G-CSF or the CXCR4 antagonist AMD3100. We found that MBL-KO animals displayed a significant decrease in the release of cells from BM into PB compared with control WT mice (19). This result provided evidence for the pivotal role of the MBL pathway in the mobilization process. However, despite a significant decrease in egress of HSPCs from BM to PB, this process was not completely inhibited, which suggests the presence of redundant pro-mobilizing mechanisms. Based on our finding that factor B deficiency in mice also impairs mobilization of HSPCs, the persistence of some level of mobilization in MBL-KO mice could be explained by parallel activation of an alternative pathway (45). This possibility is currently being investigated in more details in our laboratory.

## The Pivotal Role of the MBL Pathway of ComC Activation in Triggering Mobilization of HSPCs

**Figure 1:**
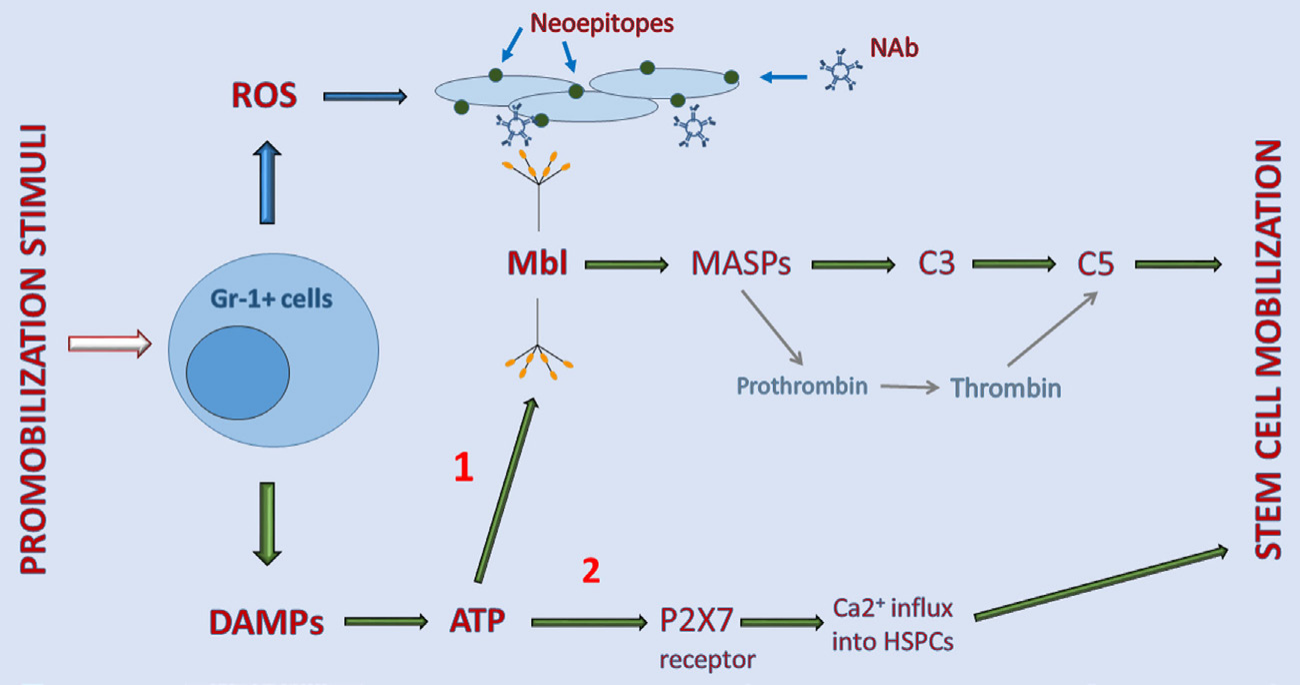
Interaction between elements of purinergic signaling and activation of the complement cascade (ComC) in the induction of sterile inflammation in bone marrow (BM). Stimulatory factors released during tissue/organ injury, systemic mediators of inflammation, and pharmacological inducers of hematopoietic stem/progenitor cells (HSPC) mobilization activate Gr-1^+^ leukocytes in BM to release danger-associated molecular patterns (DAMP) molecules, including adenosine triphosphate (ATP) and reactive oxygen species (ROS). As a DAMP molecule, ATP is recognized by MBL, which activates the ComC and CoaC in an MASP-dependent manner (*indicated on a graph as 1*). By contrast, ROS exposes neoepitopes, and neoepitope–IgM complexes are also recognized by mannan-binding lectin (MBL). This leads to activation of the ComC by the MBL-dependent pathway. Both classical C5 convertase, as a product of C3 cleavage, and C5-like convertase activity, provided by thrombin cleaving C5 to release cleavage fragments C5a and _desArg_C5a, are crucial in the egress of HSPCs from BM. In addition to serving as a DAMP (*indicated on a graph as 2*), ATP also activates purinergic receptors expressed on the surface of HSPCs, in which P2X7 plays an important role in promoting calcium influx into cells (*indicated on a graph as 2*). This facilitates intracellular actin fiber rearrangement that is crucial in cell migration and egress from BM.

Recognition of the involvement of the MBL pathway in egress of cells from BM not only further supported a crucial role of innate immunity in triggering the mobilization process but also shed more light on the cellular and molecular events regulating this process. Our understanding of this phenomenon is supported by the experimental data depicted in Figure 1.

As indicated in Figure 1, pharmacological mobilizing agents, including recombinant G-CSF, synthetic AMD3100, and natural mediators of inflammation or tissue organ/injury, such as (i) endogenous G-CSF secreted by endothelium, macrophages and immune cells, (ii) C5a and C3a released in damaged tissues, (iii) interleukin 8 (IL-8), (iv) bacteria-derived N-formylmethionyl-leucyl-phenylalanine, or (v) leukotriene B4 (LTB4) secreted from activated granulocytes, are all able to initiate the sequence of events leading to activation of the ComC (1–6). The important role of BM-residing leukocytes, which are crucial in the mobilization process, has been demonstrated by seminal papers showing that neutrophil depletion in BM negatively affects the efficiency of this process (46, 47).

Overall, activated leukocytes release several pro-inflammatory factors, including DAMPs and free radicals (ROS) (5, 48–50). DAMP molecules secreted by leukocytes include mainly ATP but also other members of this family, including high mobility group box 1 (HMGB-1) protein, heat shock proteins, and the S100 multigenic family of calcium-modulated proteins (28). What is highly relevant for the topic of this review is that ATP is the most important DAMP and is recognized by a soluble pattern-recognition receptor, MBL (19, 29, 40, 51). On the other hand, in addition to DAMPs, cells under stress release reactive oxygen species (ROS). When released from leukocytes, ROS expose neoepitope antigens on the surface of cells in the BM microenvironment that are recognized by naturally occurring antibodies, mainly from the IgM class (52). Of note in addition to ATP, neoepitope–IgM complexes are also recognized by the same MBL molecule (Figure 1).

In the next step, MBL activates mannan-binding serum proteases (MASPs) that cleave C3 and thereby trigger ComC activation in the MBL-dependent pathway (19, 33). As shown in Figure 1, MASP-1 activates the CoaC in parallel (33). Activation/cleavage of C3 creates C5 convertase, which cleaves C5 to the anaphylatoxins C5a, _desArg_C5a, and releases iC5b during the cleavage process. iC5b, in turn, is involved in generation of the membrane attack complex (C5bC9) (29, 43). Moreover, in parallel, cleavage of C5 is augmented by thrombin generated during activation of the CoaC, as thrombin is a proteolytic enzyme with C5 convertase-like activity (53). This activity explains why both the ComC and the CoaC are activated during the mobilization process (54, 55).

As mentioned above, activation of the distal part of the ComC is crucial for the egress of cells from BM. First, after the ComC is activated in the BM microenvironment, C5a and _desArg_C5a activate granulocytes that help to release HSPCs from their niches by (i) secretion of several proteolytic enzymes that disrupts the SDF-1–CXCR4 and VCAM-1–VLA-4 retention axes operating between HSPCs and the cells lining the BM niches and (ii) release of phospholipase Cβ2 that digests the GPI-A component of membrane lipid rafts, which is crucial in maintaining lipid raft integrity (38). Disruption of membrane lipid rafts negatively impacts the retention functions of the CXCR4 and VLA-4 receptors, which are membrane lipid raft-associated receptors (38). Next, the ComC activated in BM sinusoids directly chemoattracts granulocytes, which are the first cells to egress from BM into circulation. These cells are rich in proteolytic enzymes and help to disrupt the endothelial barrier and thus pave the way for HSPCs to follow behind (43). Finally, the HSPCs that are released from their niches follow a steep gradient of bioactive sphingolipids, such as sphingosine-1-phosphate (S1P) and ceramide-1-phosphate (C1P), which are present at high concentrations in BM sinusoids (5, 56–58). Both of these phosphosphingolipids are potent chemoattractants for HSPCs at the physiological concentrations present in PB (56). The gradients of both S1P and C1P are already very steep under steady-state conditions in PB and may additionally steepen due to the release of S1P form red blood cells in BM sinusoids exposed to MAC. As mentioned above, the egress of HSPCs that do not respond directly to a C5a chemotactic gradient is facilitated by granulocytes, which are the first cells to egress BM in a C5a gradient-dependent manner (43).

Besides activating MBL, ATP released from cells activates in parallel certain purinergic receptors on the cell surface that augment the mobilization process. The most important of these receptors seems to be a P2 family member, the P2X7 receptor ion channel (6, 17, 59, 60). As discussed below, P2X7 allows an influx of Ca^2+^ ions into cells that activate changes in the cell cytoskeleton that are important for cell migration and adhesion (61).

## Purinergic Signaling in BM and its Link to ComC Activation

As shown in Figure 1, ATP is an important DAMP and extracellular nucleotides (EXN) that is released from activated neutrophils, and as a DAMP, it activates the MBL pathway of the ComC, and as an EXN, it activates purinergic signaling pathways that additionally promote egress of HSPCs from BM into PB (17, 61–69).

Purinergic signaling is an ancient form of extracellular signaling mediated by EXNs, including most importantly the purine ATP and its metabolite nucleoside, adenosine (34). Purinergic signaling also involves certain rare extracellular pyrimidines, such as UTP and UDP. Purinergic receptors for EXNs are expressed on all cells in the body and are represented by several families of P1, P2X, and P2Y receptors, which are among the most abundant receptors in living organisms (34). HSPCs express several receptors that belong to two different purinergic receptor families, P1 and P2 (34). While the P1 receptor family consists of four G protein-coupled receptor subtypes, A_1_, A_2A_, A_2B_, and A_3_, which are activated by adenosine (62), the P2 family includes a total of eight receptors (P2Y1, 2, 4, 6, 11, 12, 13, and 14) identified so far, which are G protein-coupled receptors and respond to stimulation by ATP, ADP, UTP, and UDP. The P2X ionotropic channel receptor family consists of seven members (P2X1, 2, 3, 4, 5, 6, and 7), which are activated by ATP (34).

However, the main purpose of this review is to show the role of EXNs and purinergic signaling in inducing sterile inflammation in BM, which plays a role in the mobilization of cells into PB, and it is important to realize that EXNs also have pleiotropic effects in regulating hematopoiesis (59–64). For example, EXNs, particularly ATP and adenosine, have been reported to promote proliferation of HSPCs in zebra fish and murine embryos (63). By contrast, UTP has been reported to inhibit the proliferation and migration of leukemic cells. The overall role of purinergic signaling in maintaining BM homeostasis is discussed in excellent review elsewhere (64).

It has been postulated that in the induction of sterile inflammation in BM a crucial role is played by ATP, which is secreted from activated BM cells, mainly granulocytes, *via* pannexin channels as a DAMP molecule, and as we have demonstrated, pharmacological inhibition of pannexin by employing a drug (probenecid) or a specific anti-Panx1 blocking peptide decreases G-CSF- and AMD3100-induced mobilization of HSPCs (17). Connexin-43 is also involved in the release of ATP, and some ATP is also secreted in an extracellular microvesicle-dependent manner (31, 65). The involvement of connnexin 43 gap junction proteins in ATP secretion is supported by the fact that connexin-43-KO mice are poor mobilizers (65). This defect could be at least partially explained as we envision by impaired release of ATP from cells.

Based on this finding, ATP secreted by granulocytes and other BM cells is recognized as a DAMP by MBL, which triggers activation of the ComC (Figure 1). On the other hand, as depicted, ATP also interacts with P2 purinergic receptors, and the P2X7 receptor plays an especially pivotal role in mobilization. In support of this notion, we found that P2X7-KO mice are poor mobilizers (17). Moreover, studies in chimeric mice in which WT animals were reconstituted with P2X7 BM cells, and P2X7-KO mice were reconstituted with WT marrow cells revealed that this defect is due to a lack of P2X7 on the surface of hematopoietic and not non-hematopoietic cells in the hematopoietic microenvironment (17).

**Figure 2:**
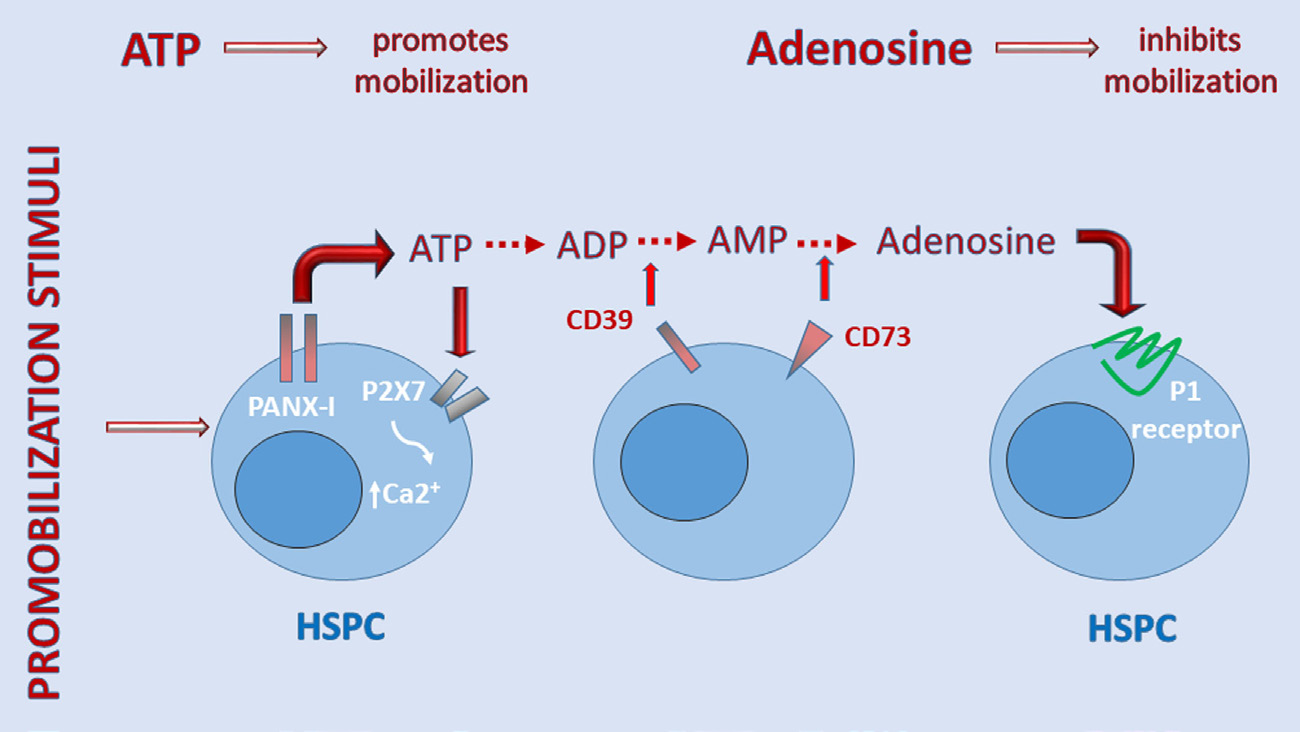
A self-limiting purinergic signaling mechanism in the induction of sterile inflammation in bone marrow (BM). Adenosine triphosphate (ATP), which is abundantly released from cells *via* pannexin channels as a danger-associated molecular pattern (DAMP), is also processed in the extracellular space by several ectonucelotidases, including cell-surface-expressed CD39 and CD73, and these are crucial to generating extracellular adenosine. While ATP promotes sterile inflammation in the BM microenvironment and mobilization of hematopoietic stem/progenitor cells (HSPCs), adenosine has the opposite effect. Inhibitors of ectonuclotidases facilitate sterile inflammation in BM and egress of HSPCs. By contrast, inhibitors of adenosine receptors are expected to inhibit this process.

Figure 2 shows that when ATP is released into the extracellular space, if it does not bind to MBL, it activates the P2X7 receptor to allow influx of Ca^2+^ or, in parallel, is converted by cell-surface ectonucleotidase CD39 to AMP, which is then converted by ectonucleotidase CD73 to the nucleoside adenosine (34, 64). The importance of the purinergic signaling cascade is further supported by our recent observation that CD73-deficient mice, which because of ectonucleotidase deficiency have less free adenosine in the extracellular space, mobilize greater numbers of HSPCs, which indicates a negative regulatory role for adenosine in the mobilization process (17). The inhibitory mobilizing effect of adenosine has been confirmed by injecting mice with this nucleoside along with pro-mobilizing agents (17). These results demonstrate that, while ATP triggers and promotes the mobilization process, adenosine generated from ATP provides negative regulatory feedback and plays an opposing inhibitory role (Figure 1).

Based on these findings, ATP triggers, on the one hand, as a DAMP, activation of the ComC in an MBL-dependent manner and, on the other hand, regulates the mobilization process in a more complex way by activating P2 receptors and providing negative feedback control mechanisms for this process through its metabolite, adenosine, which engages P1 receptors on the surface of cells (17, 62, 64).

What is also important is to realize that purinergic receptors are expressed on the surface of several types of cells that comprise innate immunity cellular components, including granulocytes, basophils, eosinophils, monocytes, and dendritic cells as well as cells in the BM microenvironment, including stromal cells, osteoblasts, osteoclasts, pericytes, and endothelial cells (64). ATP is also released from the synapses of neural fibers innervating BM tissue (34). Furthermore, the C3 and C5 cleavage fragment receptors, C3aR and C5aR, respectively, are expressed by several hematopoietic and non-hematopoietic cells in the BM microenvironment (41–43). All these add significant complexity to the relationship between purinergic signaling and innate immunity in BM and requires further study.

Moreover, it is important to pin point that extracellular ATP also exerts strong pro-inflammatory effects that are independent from MBL activation (28). ATP may also activate NLRP3 inflammasome pathway in cells that controls in caspase-1-dependent manner maturation of two important pro-inflammatory members of interleukin (IL-1) family cytokines—namely IL-1β and IL-18 (67–69). It has been postulated that activation of the NLRP3 inflammasome is regulated at both the transcriptional and post-translational levels. While the transcription of inflammasome is induced by the signal mediated by toll-like receptor/nuclear factor-kB pathway, NRLP3 inflammasome activation at post-translational level is mediated e.g., by various DAMPs including ATP. Both IL-1β and IL-18 are released from cells in response to ATP-mediated activation of NLRP3 inflammasome and potentiate state of sterile inflammation in BM microenvironment that promotes mobilization process.

## Purinergic Signaling, Innate Immunity, BM Sterile Inflammation, and Hematopoietic Aging

Aging is an inevitable and complex process involving a sequence of pathological events (22, 23, 66). Several mechanisms are currently being proposed that accelerate this process at the cellular level, including shortening of the tips of chromosomes (telomeres); generation of ROS, which contribute to replication stress and oxidative DNA damage; impairment over time of the process of autophagy, a major degradation pathway essential for removing damaged organelles and macromolecules from the cytoplasm in eukaryotic cells, and which promotes recycling of amino acids during periods of starvation; the occurrence of pathologic lipid metabolism; and chronic inflammation (22, 66).

Aging also occurs in the hematopoietic system and is characterized by a myeloid bias, in which BM increases the number of myeloid progenitors along with impaired differentiation of these cells, gradually developing anemia and decreasing the number and fitness of B cell progenitors, which is accompanied by oligoclonal expansion of memory B and T cells (22, 23). Additional evidence indicates that this process is triggered by low-grade chronic inflammation that is a result of an increase in activation of innate immunity and impaired acquired immunity (22, 23). These changes may lead to the appearance of BM myelodysplasia and, in consequence, to clonal cell expansion and overt leukemia (22). These changes also lead to an increased incidence of autoimmunological diseases that are observed in patients with advancing age (22, 23, 66).

Since aged cells in the BM microenvironment are a rich source of DAMP molecules, including mostly ATP and HMGB-1, one can speculate that low-grade chronic inflammation in the BM microenvironment may be triggered by chronic activation of the ComC (6). A similar mechanism is also most likely involved in the aging of other organs, such as heart, brain, or kidney. This suggests that an effective countermeasure to ameliorate this unwanted effect would be anti-inflammatory treatment.

To shed more light on this phenomenon, there is a need for more long-term studies in ComC-deficient mice to see whether these animals are protected from age-related dysfunction of vital organs. The earlier study showing that C3-deficient mice fail to display age-related hippocampal decline lends support to undertaking more complex studies (70). In particular, it would be interesting to see whether MBL-KO mice or C5-KO mice are endowed with an extended life span and develop fewer age-related pathologies in vital organs. Such experiments, of course, should prevent exposure of these animals to potential pathogens, as their susceptibility to infection may affect the final long-term experimental results.

## Innate Immunity as a Potential Trigger of GvHD

Graft-versus-host disease is a serious medical complication seen in patients who are recipients of transplanted tissue or cells from a genetically histoincompatible donor (25, 71). GvHD is commonly observed after hematopoietic stem cell transplants, when T cells present in the graft attack the tissues of the transplanted recipient. After perceiving host tissue antigens, among them the human leukocyte antigens, as antigenically foreign, T cells produce an excess of cytokines, including tumor necrosis factor alpha (TNFα) and interferon gamma (IFNγ) (25, 27, 71).

An important question remains: To what degree are innate immunity, in particular the ComC, and purinergic danger signaling involved in triggering this T-cell-mediated process? Unfortunately, conclusive experiments in animal models have not been performed. However, a very recent report indicates that patients with defects in activation of the MBL pathway of ComC activation are partially protected from this so often devastating transplant complication (25). It would be interesting to see whether MBL-KO mice are more resistant to GvHD after allogeneic BM transplants than their WT littermates.

## Therapeutic Implications for Modulating Sterile Inflammation in BM

While activation of the ComC is important for optimal mobilization, its inhibition is highly relevant to ameliorating the chronic, sterile inflammation process seen in aging and myelodysplasia. Inhibition of the ComC may also be of importance in ameliorating the onset of GvHD, which occurs after infusion of histoincompatible hematopoietic cells.

Overall, since ATP-mediated activation of the MBL pathway of ComC activation leads to induction of sterile inflammation in the BM microenvironment, an anti-inflammatory treatment may have the opposite effect. However, this process, as depicted in Figure 2, tends to be somewhat self-limiting due to ATP conversion to adenosine. In fact, adenosine is known in immunology as an anti-inflammatory nucleoside (72). Therefore, appropriate activators of adenosine receptors would help to control sterile inflammation in the BM microenvironment. However, in proposing such a treatment, one would have to take into consideration the fact that adenosine is a powerful cardiovascular mediator, and a hyperphysiological dosage of adenosine mimetic may lead to cardiac complications (34). Moreover, taking into consideration the involvement of the P2X7 receptor in activating sterile inflammation in BM (6, 17), it would be important to test whether specific inhibitors of this receptor could be employed as potential anti-inflammatory drugs to dampen sterile inflammation in BM.

Another recently identified inhibitor of stem cell mobilization is heme oxygenase 1 (HO-1) (73–78). This anti-inflammatory enzyme, which is induced by oxidative stress in the BM microenvironment, counteracts the induction of sterile inflammation in BM. We provided evidence that HO-1 is a potent inhibitor of hematopoietic cell migration and the responsiveness of HSPCs to crucial chemoattractants, such as S1P, C1P, and SDF-1 (72, 78). Moreover, mice that lack HO-1 are easy mobilizers (72). The biological anti-inflammatory and ComC-activation properties of HO-1 have been demonstrated both in HO-1-deficient mice and in a case of rare human HO-1 deficiency in which the ComC became continuously hyperactivated (77). This hyperactivity of the ComC related to HO-1 deficiency leads to chronic inflammation in affected individuals. In our most recent work, we demonstrated that ATP and adenosine directly modulate expression of HO-1 in hematopoietic cells (17). While ATP inhibits HO-1 expression at the mRNA level, adenosine, by contrast, upregulates HO-1 mRNA expression. These results correspond with the opposing effects of ATP and adenosine on the mobilization process. Therefore, based on the results cited above, small-molecule activators of HO-1 could be employed to control sterile inflammation in the BM microenvironment.

By contrast, inhibition of adenosine generation in the BM extracellular space, for example, by employing inhibitors of the ectonucleotidase CD73 or downregulation of HO-1 expression in the BM microenvironment by employing small-molecule HO-1 inhibitors, should promote the onset of sterile inflammation. This would be beneficial for facilitating egress of hematopoietic cells into the circulation to harvest more HSPCs for transplantation (17, 72). In support of this possibility, our recent study showed that CD73-KO mice, which do not convert AMP to adenosine in the extracellular space, are in fact easy mobilizers of HSPCs (17). Other potential targets for facilitating this process are inhibitors of other ectonucleotidases, such as CD39, or even a P2X7 receptor mimetic.

## Conclusion

In this review, we presented the concept that sterile inflammation in the BM microenvironment is involved in the egress of hematopoietic cells, including HSPCs, into the circulation (6, 17). We also presented a novel link between activation of purinergic signaling and the release of EXNs, mainly ATP, which is a crucial activator of the MBL pathway of ComC activation. We are aware that purinergic signaling and EXNs play pleiotropic roles in modulating the activity of the innate and acquired immune systems, but our recent results highlight the importance of ATP as a DAMP molecule in triggering the mobilization process. A similar mechanism regulating the egress of cells from BM into PB is probably also involved in the egress of cells into lymphatics (3, 6). Besides HSPCs, the interplay between purinergic signaling and innate immunity also plays a role in mobilization of lymphocytic progenitors and other types of stem cells, including MSCs, EPCs, and VSELs, and this is currently being investigated in our laboratory.

Functional P1 and P2 purinergic receptors are expressed on the surface of several types of non-hematopoietic cells in the BM microenvironment (64, 79), including cells forming stem cell niches, such as perivascular SDF-1^+^ and KL^+^ mesenchymal stromal cells and endothelial cells as well as cells in quiescent nestin^bright^ NG2^+^ arteriolar and proliferative nestin^dim^Lepr^+^ sinusoidal niches. This wide distribution of these receptors opens up a new area of investigation to better understand the complexity of stem cell mobilization and to design optimal mobilization strategies. Purinergic receptors are also expressed by osteoblasts lining trabecular bones as well as osteoclasts (64). Finally, ATP may also be involved as a neurotransmitter, in addition to catecholamine, in neural fibers that innervate BM tissue in modulating β-adrenergic-mediated egress of HSPCs from BM niches (12, 34).

On the other hand, it is important to better understand the role of sterile inflammation in the aging of hematopoietic cells and its potential involvement in myelodysplasia and GvHD. Shedding more light on these phenomena will also allow us to develop more efficient treatment strategies. Purinergic signaling in both steady-state hematopoiesis and pathology has become an exciting field of investigation.

## Author Contributions

This manuscript was written by MR in consultation with the rest of the authors. All authors approved the manuscript.

## Conflict of Interest Statement

The authors declare that the research was conducted in the absence of any commercial or financial relationships that could be construed as a potential conflict of interest.
